# The Intersection of Human and Veterinary Medicine—A Possible Direction towards the Improvement of Cell Therapy Protocols in the Treatment of Perianal Fistulas

**DOI:** 10.3390/ijms232213917

**Published:** 2022-11-11

**Authors:** Anna Burdzinska, Marek Galanty, Sabina Więcek, Filip A. Dabrowski, Ahmed Lotfy, Tomasz Sadkowski

**Affiliations:** 1 Department of Immunology, Transplantology and Internal Diseases, Medical University of Warsaw, 02-006 Warsaw, Poland; 2 Department of Physiological Sciences, Institute of Veterinary Medicine, Warsaw University of Life Sciences, 02-776 Warsaw, Poland; 3 Department of Small Animal Diseases and Clinic, Institute of Veterinary Medicine, Warsaw University of Life Sciences, 02-776 Warsaw, Poland; 4 Department of Paediatrics, Medical University of Silesia, 40-055 Katowice, Poland; 5 Department of Obstetrics, Perinatology and Neonatology, Center for Postgraduate Medical Education, 01-809 Warsaw, Poland; 6 Club35, Polish Association of Obstetricians and Gynecologist, PTGiP, 02-677 Warsaw, Poland; 7 Department of Surgery, Medical University of South Carolina, Charleston, SC 29425, USA; 8 Biotechnology and Life Sciences Department, Faculty of Postgraduate Studies for Advanced Sciences (PSAS), Beni-Suef University, Beni-Suef 62511, Egypt

**Keywords:** mesenchymal stromal cells, cell transplantation, perianal Crohn’s disease, anal furunculosis, dogs, perianal fistula, “One Health” approach, immune-mediated diseases, companion animals, large animal model

## Abstract

The effective treatment of perianal fistulizing Crohn’s disease is still a challenge. Local administration of mesenchymal stromal cells (MSCs) is becoming a part of accepted treatment options. However, as a fledgling technique, it still can be optimized. A new trend in translational research, which is in line with “One Health” approach, bases on exploiting parallels between naturally occurring diseases affecting humans and companion animals. Canine anal furunculosis (AF) has been indicated as condition analogous to human perianal Crohn’s disease (pCD). This narrative review provides the first comprehensive comparative analysis of these two diseases based on the published data. The paper also outlines the molecular mechanisms of action of MSCs which are likely to have a role in modulating the perianal fistula niche in humans, and refers them to the current knowledge on the immunomodulatory properties of canine MSCs. Generally, the pathogenesis of both diseases shares main determinants such as the presence of genetic predispositions, dysregulation of immune response and the relation to intestine microbiota. However, we also identified many aspects which should be further specified, such as determining the frequency of true fistulas formation in AF patients, elucidating the role of TNF and Th17 pathway in the pathogenesis of AF, or clarifying the role of epithelial-to-mesenchymal transition phenomenon in the formation of canine fistulae. Nevertheless, the available data support the hypothesis that the results from testing cell therapies in dogs with anal furunculosis have a significant translational value in optimizing MSC transplants procedures in pCD patients.

## 1. Introduction

Crohn’s disease (CD) is a chronic, recurrent condition with a complex and not fully understood pathogenesis mostly related to the defects of immune regulation. The perianal form of CD (pCD) is one of the most burdensome for patients and most difficult to treat. Patients with pCD present a range of manifestations such as skin tags, fissures, ulcers, strictures, abscesses, and fistulas [1]. These anorectal pathologies constitute not only medical, but also substantial social problems, leading to a significant reduction in the quality of life [2]. The management of patients with pCD, and especially those presenting with multiply perianal fistulas, is challenging and includes complex pharmacotherapy often combined with surgical interventions [3]. Despite the significant improvement of therapeutic possibilities related to the introduction of biological drugs such as anti-TNF (anti-tumor necrosis factor), the rate of treatment failure and relapse in patients with pCD is still relatively high [4]. Additionally, prolonged immunosuppressive therapy and repeated surgical interventions expose patients to serious adverse effects. Therefore, it is reasonable to look for new therapeutic strategies that could replace or support those currently used in the treatment of pCD.

## 2. Local Cell Injections as Treatment of Fistulizing Crohn’s Disease

Within recent years, one of the most promising new approaches in managing perianal fistulas seems to be the transplantation of mesenchymal stem/stromal cells (MSCs). The authors of the most recent meta-analysis including all together over 1200 patients demonstrate that the local administration of MSCs causes less adverse events and is more effective than procedures performed in control groups [5]. In the biggest currently available randomized, double-blind, placebo-controlled study, which enrolled 212 patients with pCD, MSCs were locally injected around perianal fistulas with a follow-up period of 24 weeks [6]. In this trial, healing (defined as closure of all external openings that were draining at baseline, and absence of collections >2 cm of the treated perianal fistulas confirmed by masked central MRI) was achieved in 50% of patients from the MSC group (88 patients completed the follow-up) and 34% of patients from the placebo group (83 patients completed the study). The difference between groups was statistically significant. It is worth mentioning that majority of patients (from both arms of the study) were subject to concomitant standard pharmacotherapy in the course of the trial. Meta-analysis of results coming from four randomized clinical trials reports that the median proportion of patients with improved total external healing was 42% in MSC groups (comparing to 25% of patients from control arms) [7]. These data are generally very promising suggesting the appearance of a novel, minimally invasive and relatively effective approach in the treatment of perianal fistulas in CD. They formed the basis for the first and so far the only MSC-based product registration for therapeutic use in Europe, which is indicated for the treatment of complex anal fistulas in adult patients with inactive/poorly active luminal Crohn’s disease. Moreover, recent analysis based on economic modeling indicates that MSC-based therapy (with cell preparation performed in academic good manufacturing practice grade facilities) can be more cost-effective than other current pCD treatment approaches such as fecal diversion [8]. Nevertheless, there is an agreement that many aspects regarding MSC-based procedures could be improved. Issues that certainly may be further optimized are cell source, cell dose, preconditioning methods or genetic manipulations, defining predictive markers of clinical response from both the transplanted cell population and the patient. Clearly, these steps should be performed with the support of preclinical studies. Fistulizing Crohn’s disease is a very difficult condition to mimic in an animal model. To date, very few rodent models of fistulae have been proposed [9]. These models can be important tools for testing hypotheses on early stages in the path to clinical trials [10] or in understanding the pathogenesis of the disease [11]. However, they have certain limitations such as short-term tested durability (i.e., 7 days [12]), the need of performing technically advanced human–mice xenografts [13] or using animals with fully functional immune system [10,12], which does not correspond to the dysregulated immune mechanisms found in CD patients. Moreover, testing the effects of cell therapies in standard small animal models is considered to be of rather poor predictive value for subsequent clinical outcomes in humans [14]. Although new and interesting approaches are presented, such as predictions of clinical results for new drug candidates using mouse-transcriptome database ‘‘humanized’’ by machine learning algorithms and human clinical trials datasets [15], such strategies are unlikely to be applicable in the prognosis of the effects of cell-based therapies in the near future. Therefore, searching for other preclinical options which would allow to accurately forecast the effectiveness of new therapeutic solutions in patients with perianal fistulas is still justified.

An interesting approach in translational research is the concept of exploiting the similarities between diseases affecting humans and companion animals [16,17]. Our four-legged friends are influenced by civilization factors, which include highly processed food, exposure to chronic stress, lack of exercise, low-diversity mucosal microbiota, air pollution, and others. Not surprisingly, dogs and cats are diagnosed with many diseases that are very similar to those found in humans—they have a corresponding pathogenesis, course, and response to treatment. Conditions related to the dysfunction of the immune system occupy an important place here [17]. Dogs can develop, for example, inflammatory bowel disease (considered a model of IBD in humans), dry conjunctivitis, and keratitis (considered a model of Sjögren’s syndrome), atopic dermatitis, pemphigus deciduous, lupus erythematosus, type I diabetes, as well as anal furunculosis (considered a model of perianal fistulas in CD disease). The later condition is the focus of this article. It has been postulated that testing the effects of cell transplantations in companion animals with naturally occurring human-like diseases may provide bi-directional benefits: contribute to the development of new therapies for veterinary medicine and create unique predictive values for the outcome of human clinical trials [17]. With regard to perianal fistulas, only one report demonstrates the effect of local MSC transplantation in naturally affected dogs [18]. In this study, human-embryonic-stem-cell-derived MSCs were injected around the perianal sinuses in six dogs with AF. The procedure was well tolerated, and at 3 months all dogs were completely free of fistulas (the concomitant pharmacological treatment was maintained for at least 30 days after cell transplantation). These results, although very preliminary, encourage further research in this area. Of course, testing new therapies in companion animals must be conducted in accordance with the medical art and local ethical regulations. Additionally, such trials must be accompanied by full awareness that dogs enrolled to the study are veterinary patients and not experimental animals. A “primum non nocere” basis must be a priority in this kind of studies. Thus, the use of companion animals for testing new therapies should only be done when there are already strong indications that the procedure is not harmful and potentially beneficial. MSC transplantations meet both of these conditions. This kind of research is in agreement with One Health perspective—a collaborative, transdisciplinary approach aimed at achieving optimal health outcomes recognizing the interconnection between people, animals, and their shared environment [17,19]. However, as pointed out by Kol et al., the identification of the naturally occurring disease “model” in companion animals for a given human disease requires in-depth comparative analysis of numerous issues such as anatomy, pathology, clinical manifestations and response to treatment [16]. Although canine anal furunculosis has been indicated several times as a condition analogous to human fistulas in Crohn’s disease [9,17,18,20], a comprehensive, multi-layered juxtaposition of these two diseases has not been presented in the literature. This type of comparative survey based on the available data on canine AF and human pCD is the main focus of the present article.

## 3. Anatomy of Canine and Human Perianal Region

**Figure 1:**
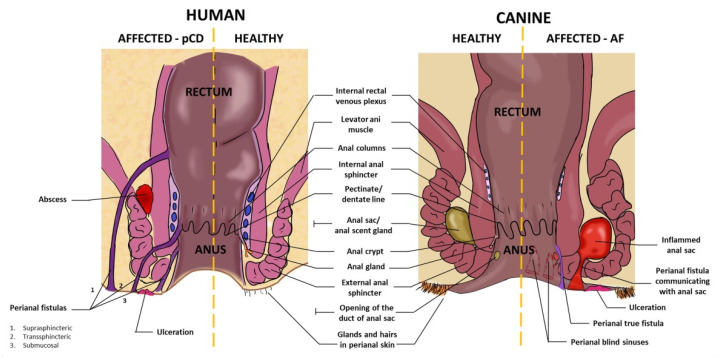
The schematic longitudinal cross-section of human and canine perianal regions. One side of each picture (healthy) presents the appearance of normal perianal area in both species and the other (affected) shows the same regions with most important pathological elements associated with human perianal Crohn’s disease (pCD) and canine anal furunculosis (AF).

In humans and dogs, the anal canal is surrounded by the internal anal sphincter (smooth muscle) and the external anal sphincter (skeletal muscle). The distal (cutaneous) part of the anal canal is covered by the stratified squamous epithelium (anoderm) which then turns cranially into the single columnar epithelium, the lining of the colorectal part of the gut. This transition point is called the dentate or pectinate line. Cranially to the dentate line, the mucosa form longitudinal folds (columns) [21,22]. All of the above elements of anatomy are very similar in humans and dogs. The most important anatomical elements of the perianal area of dogs and humans are presented in Figure 1. Importantly, from a translational point of view, the size of the described structures in dogs (especially large breeds) is comparable to those in humans. Canine anal furunculosis is particularly common in German Shepherds (GSD), which weigh around 30–40 kg. Secretory structures are another important element of the perianal anatomy. In humans, there are several (3–10, median 6) merocrine anal glands which enter crypts formed in the distal base of the mucosal columns. The terminal branches of some anal glands end up in the submucosa, and some of them (15 to 50%, depending on the study) extend to the internal anal sphincter [23,24]. In dogs, analogous glands (called “true” anal glands) were also described, but their number and extension have not been reported in detail. In the perianal skin there are circumanal glands (in dogs called also hepatoid glands), another structure common to both species. These are sebaceous, or less commonly, apocrine glands, with ducts opening into the hair funnels [25]. However, there also differences in anatomical aspects of the perianal region between analyzed species. One of the most important refers to the presence of anal scent glands, called anal sacs. These glands are present in dogs, similar to other carnivores and some other mammals, but not in humans (at least not in their fully developed, functional form) [24]. Anal sacs are big, paired, well-defined anatomical structures containing modified sweat apocrine and sebaceous glands. They lie ventrolateral in between internal and external anal sphincters. The emptying ducts open to the anal canal caudally to the dentate line. The secretions of anal sacs play role in the chemical communication between individuals [26]. The inflammatory processes in both anal sacs in dogs and anal glands in humans can contribute to the formation of perianal abscesses and fistulas. Another difficult-to-overlook anatomical difference between the perianal region of humans and dogs is the tail. Interestingly, in the previous century it was postulated that low tail carriage (like it is in German Shepherds) favours faecal retention in warm and humid environment which can lead to local chronic inflammation and subsequently to canine anal furunculosis [27]. Today, this theory is no longer valid [20]; therefore, the lack of a tail in humans is not so meaningful in the analyzed issue. The current views on the canine AF etiopathogenesis will be discussed in the following paragraphs.

## 4. Prevalence, Predispositions and Association with the Inflammation of the Intestine

Crohn’s disease is, next to ulcerative colitis, the form of inflammatory bowel disease (IBD). The IBD is diagnosed mainly in middle-aged adults (with slightly higher prevalence in women in majority of studies), but recently it has been frequently discovered in children, even during infancy (no sex predisposition reported in pediatric patients) [28]. Canine anal furunculosis is also diagnosed mainly in middle-aged individuals. The incidence of AF in a dog younger than 1 year was mentioned only in one report [29]. The occurrence of canine AF was not shown to be associated with gender so far, but there is a strong, well-proven breed predisposition. About 85% of dogs diagnosed with anal furunculosis are German Shepherds [30,31,32], which suggest that genetics is a significant determinant in the pathogenesis of this disorder. The association of perianal CD with luminal form of a disease in humans has been established [33]. The cumulative incidence of perianal Crohn’s disease within the population of patients with CD was estimated at nearly 20% in 10 years follow-up (data from 12 population-based studies, mainly European) [34]. Only 5% of patients presenting with pCD had no evidence of luminal abnormalities [1]. The association of perianal manifestations with intestinal inflammation in dogs is not as clear as in humans. It was analyzed only in one study on 18 dogs with AF diagnosis [30]. Histological signs of colitis (inflammatory mucosal changes in multiply biopsy specimens) were recognized in 9 dogs (50%) of which only one case was classified as moderate, and the others were considered mild. Additionally, in two dogs with no histological signs of colitis, the proctitis was observed in endoscopic evaluation (11%). Other fragments of the intestine have not been assessed. Clinical signs typical for colitis (diarrhoea, mucus in faeces) were observed with the same frequency in patients with and without histological diagnosis of mucosal inflammation [30]. Based on this study, there is a probable link between canine AF and colitis (or wider—inflammation of any fragment of the intestine), but this requires further confirmation.

## 5. Local Clinical Manifestations of Perianal CD in Humans and Anal Furunculosis in Dogs

In humans, clinical signs of perianal Crohn’s disease include various tissue destruction, such as fissures, skin tags, and ulcerations. Another type of manifestations are anorectal strictures, which are related to the chronic inflammation of anal canal and rectum associated with long-term remodeling of extracellular matrix. The last and most troublesome category of symptoms includes fistulas and abscesses (Figure 1). Anal fistula is defined as abnormal connection (canal) between two epithelialized surfaces, most classically between anorectum and perianal skin. Therefore, it has two openings—internal and external in the classic fistula. Fistulas differ in their course and the location of the openings, and are subject of various classification systems in the clinical practice. Perianal fistulas are often multiplied and complex. They constitute the most difficult challenge in the treatment of pCD [35].

In dogs, anal furunculosis manifests mainly with ulcerations and the presence of sinuses in the perianal area. The sinuses may have blind ends, or they can communicate with either anal sacs or anorectal lumen (presented in Figure 1). It is mentioned in the recent review [20] that true fistulas linking perianal skin with epithelialized surface of anorectum are rather rare in dogs. Surprisingly, the literature lacks clear data on the proportion of dogs with AF presenting with true anal fistulas. Only in one study, authors divided patients with anal furunculosis (*n* = 40) into groups based on the dominating clinical manifestation—eleven dogs presented with deep sinuses frequently becoming true fistulas, twenty dogs had extensive but more superficial ulcerations, whereas in the remaining nine dogs, both types of lesions were coexisting [36].

## 6. Clinical Response to Pharmacological Treatment

Comparative analysis of diseases cannot be complete without looking at the treatment regimens used and their effectiveness. Overall, the current recommendations for both canine AF and human pCD include a combination of pharmacotherapy and surgery. The comprehensive descriptions of the treatment options for both diseases were reviewed elsewhere [20,37]. Herein, we focus on the aspects of pharmacological treatments which can help identify similarities/differences in pathogenesis of both conditions. The most important points of this comparison are summarized in Table 1.

In dogs, up to the mid-1990s, surgical interventions were the primary and often the only treatment for anal furunculosis [52]. It was already recognized at that time that antibiotics alone do not result in satisfactory healing of perianal sinuses in dogs; however, no data regarding this issue have been published [20]. A shift in the treatment of canine anal furunculosis was pioneered by Mathews and colleagues who for the first time (in 1997) reported the results of oral Cyclosporin A (CsA) administration in dogs with AF [53]. The earlier literature had already mentioned the similarities between canine AF and human pCD [32]. In the first half of the 1990s, two reports suggested that intravenous CsA infusions may be effective in the treatment of anal fistulas in patients with CD [54,55]. Matthews and colleagues clearly acknowledged that these data were the stimulus to test CsA in dogs with AF. In a small (20 patients in total), but placebo controlled, veterinary clinical trial it was distinctly demonstrated that treatment of canine AF with CsA is highly effective—all dogs treated with cyclosporine and none of the dogs treated with placebo improved after 4 weeks [38]. The healing rate in CsA treated animals at 16 weeks was 85%, but 40% of patients relapsed after treatment discontinuation. Similar response to CsA administration was later reported by several other studies (none of them included a control group), the largest of which enrolled 26 dogs [56,57,58]. Since then, CsA remains the first line treatment for AF in dogs (if the pet owner can afford it). Interestingly, in human medicine, eventually there was no studies which would definitely confirm the efficacy of CsA in the treatment of perianal fistulas. Therefore, the current treatment recommendations for pCD do not include this drug [59]. Cyclosporin is a well-known potent immunosuppressive drug that acts by inhibiting calcineurin, a factor which is activated in T cells following antigen recognition and induces (via transcription factor NFAT) the expression of cytokines related to T-cell mediated immunity. The significant inhibition of interleukin (IL)-2, the cytokine necessary for survival, proliferation, and differentiation of T cells, is a known effect of CsA treatment both in humans and dogs [60,61]. Therefore, high efficacy of CsA with concomitant poor response to antibiotics in the treatment of perianal fistulas in dogs clearly indicated the importance of inappropriate T-cell-mediated response in the pathogenesis of this condition.

In humans, the approaches to the medical treatment of fistulizing CD have also evolved within last decades. Due to the high anatomical diversity of fistulas and varied course of a disease in patients with perianal CD, treatment regimens also differ between patients. The main aim of therapy is to close the fistula tract or significantly improve the local condition. Single treatment method is often not enough to achieve this goal, so a combination of different approaches is usually necessary.

Systemic antibiotics administered for 6–8 weeks appeared to be associated with a clinical improvement of patients with pCD, although the available data are based on a limited number of studies [62]. Among the antibiotics used in the treatment of perianal fistulas, metronidazole and ciprofloxacin deserve special attention. These drugs have been shown to reduce the drainage of perianal fistulas, but not to induce their healing; therefore, they are recommended only as adjunctive therapy [63,64,65].

Thiopurine derivatives are another element therapeutic algorithms recommended in the treatment of human pCD. The thiopurine compounds are a group of antimetabolites that structurally resemble endogenous purines interfering with DNA, RNA and protein synthesis, resulting in repression of the immune response [66]. The effect of thiopurine derivatives on fistula healing in humans is considered to be moderate; however, the algorithm presented in the guidelines includes treatment with these agents in combination with antibiotics and biological drugs [39,59]. Data regarding the efficacy of thiopurines in the treatment of canine AF are very limited. The effect of monotherapy (azathioprine) was tested in one study on 13 dogs [40] and combined therapy with azathioprine and metronidazole was tested in a study on 5 dogs [67]. The reports show moderate results, usually requiring additional surgical intervention.

In humans, an important part of pharmacological regimens in the treatment of perianal CD are biological agents with anti-TNF antibodies being in the center if interest. Infliximab, one of anti-TNF used for many years in the treatment of Crohn’s disease, has been also proven to be effective in the treatment of fistulas and is currently recommended as first-line drug for both inducing a response and remission in symptomatic pCD [41]. TNF is a pleiotropic cytokine with well-known pro-inflammatory activity. Uncontrolled production or disturbed function of TNF has been linked to the development of inflammatory diseases including Crohn’s disease with its perianal form [68]. Surprisingly, the role of TNF in the pathogenesis of canine AF has not been established till now. In one study, the in vitro response of peripheral blood mononuclear cells (PBMCs) to the treatment with calcineurin inhibitors (CsA and tacrolimus) was tested. The results demonstrated that both drugs markedly inhibited *IL2*, *IL4* and *IFNG* gene expression in canine cells, similar to that in humans. However, the *TNF* expression was barely inhibited by CsA [69] (according to the general guidelines, gene symbols are italicized, and protein symbols are not italicized in this paper). These results might be an important clue in assessing the role of TNF in the development of perianal fistulas in dogs. Since CsA does not inhibit TNF in in vitro tests and is effective in the treatment of AF, it can be assumed that TNF may not be of key importance in the pathogenesis of AF in dogs as in CD in humans. However, as data indicating poor inhibition of TNF by CsA come from only one study performed on PBMCs from 3 dogs, this issue definitely requires further research.

To summarize, the pharmacological treatment of both canine AF and human pCD bases on immunomodulatory treatment with adjunctive antibiotic therapy. However, the medical approaches in both species differ with regard to the details. In dogs, the first line of treatment is cyclosporin, which is not included in pCD algorithms. In turn, in humans anti-TNFs have become the cornerstone of therapy for pCD, and these agents are neither available nor tested in dogs.

## 7. Pathogenesis of Human Perianal CD and Canine AF

Although the etiopathogenesis of human IBD is not fully known, intensive research in recent years has contributed to a significant progress in understanding mechanisms leading to the development of this disease. Very generally, they include genetic susceptibility, intestinal microbiota, environmental factors, and immunological disturbances [70].

The pathogenesis of perianal Crohn’s disease, as a subunit of IBD, covers all of the above-mentioned categories, but additionally comprises elements that are specifically involved in the formation of perianal fistulas. These include the phenomenon of epithelial-to-mesenchymal transition (EMT) and disturbed activity of the enzyme-degrading extracelullar matrix, in particular, matrix metalloproteinases (MMPs) [37,51]. The detailed presentation of all the mechanisms that have been proposed as elements of IBD (including pCD) pathogenesis is far beyond the scope of this article. Instead, existing reports on the pathogenesis of anal furunculosis in dogs will be described and referred to the published data about the pathogenesis of pCD in humans. The most important points of this comparative analysis are summarized in Table 1.

### 7.1. Intestinal Microbiota

The mucosa of the gastrointestinal tract is heavily colonized by a wide range of commensal microorganisms. The gut microbiome is necessary for the maintenance of intestinal homeostasis and broadly understood proper immunological status of the host. Commensals and pathogens share molecular patterns which are recognized by innate immunity cells. This results in a continuous stimulation of the immune system with simultaneous activation of the immune tolerance mechanisms. The loss of balance in these interactions contributes to the development of inflammatory bowel disease both in humans and in dogs [70,71]. One of the recent studies aimed to characterize the intestinal microbiome of dogs with anal furunculosis in comparison to the healthy ones [48]. The analysis was conducted on 70 fecal samples collected from 12 dogs with AF and 38 samples from 8 healthy dogs. The study based on sequencing of DNA isolated from naturally passed feces. The analysis included the filtering of host DNA and performing metagenomic profiling to determine the content of foreign DNA. The results indicate that in terms of gut commensals dogs with AF represent two groups: very dissimilar to healthy individuals (dysbiotic) and similar to healthy individuals (non-dysbiotic or healthy-like). However, in more detailed analyzes, the microbiome of “non-dysbiotic” dogs also differed significantly from healthy ones which confirms the general association of gut microbiota with the pathogenesis of canine AF. Additionally, dysbiotic dogs tended to have more severe perianal manifestations than non-dysbiotic (although the correlation was not statistically significant in this study). Interestingly, a very similar dichotomy of microbiome into dysbiotic and non-dysbiotic was reported in patients with Crohn’s disease in a significant study being a part of the Human Microbiome Project [47]. Moreover, the analysis of microbial metagenomes revealed that canine gut microbiome is significantly closer to that of humans than the other non-human microbiomes similar to that of pigs or mice [72]. The overlapping of the human and domestic dogs’ microbiomes is believed to be associated with a long-term sharing of a wide range of environmental factors [71]. Overall, the similarities of human and canine gut microbiota—both in health and disease—strongly increase the translational value of studies in dogs with anal furunculosis.

### 7.2. Dysregulation of Immune Mechanisms

Since cyclosporine was found to be highly effective in the treatment of canine AF, it has become clear that this is an immune-related disease. Several studies have been conducted in order to characterize and elucidate the role of the immune response in the development of perianal sinuses in dogs. In the 1990s, the histological landscape of perianal region in dogs with AF was described based on the analysis of biopsies collected from five defined perianal locations from 305 affected dogs [32,73]. Not surprisingly, the most heavily inflamed were sinus tracts, which were followed by anal sacs. The circumanal glands and non-ulcerated haired perianal skin displayed mild or moderate features of inflammatory process in the vast majority of cases. The external anal sphincter remained unaffected in almost all cases. Unfortunately, neither anoderm nor rectal mucosa were assessed in these studies. In all the inflamed regions, lymphoid cells constituted the main component of the infiltration. These were plasma cells expressing IgG, followed by T cells and IgA- and IgM-expressing plasma cells. In the sinus tracts, macrophages and neutrophils were also abundant; however, anal sacs samples contained macrophages, but only occasionally neutrophils. In a human study analyzing the histology of perianal fistulas in Crohn’s disease, it was similarly shown that lymphocytes (CD20+ B cells and memory CD45RO+ T cells) were dominating in the inflammatory infiltrate, next to neutrophils associated with an acute response [74]. Interestingly, it was additionally shown that cellular inflammatory composition of perianal fistulas from Crohn’s disease is markedly different than the one found in fistulas from non-Crohn’s disease as the latter contained more macrophages than lymphocytes. No direct comparative study including human and canine samples was published.

To further characterize the inflammatory process related to AF, the cytokine profile in the tissue surrounding anal sinuses was analyzed in one study involving 15 affected dogs and two control groups. The authors noted a significant increase in gene expression of pro-inflammatory cytokines, such as *IL1B*, *TNF*, *IL6*, *IL8*, and anti-inflammatory factors, such as *IL10* and *TGFB* in biopsies from perianal lesions of AF patients compared with control samples (obtained from skin wound resections unrelated to AF) [31]. These data are consistent with the cytokine profile identified in the rectal mucosa of humans with pCD [75], but unfortunately, as an isolated piece of information, this does not say much about the character of the inflammatory process in AF. However, the authors [31] additionally demonstrated a robust induction of *IL2* and *IFNG* expression with concomitant absence of *IL4* mRNA in nearly all the biopsies taken from anal furunculosis lesions, which clearly indicates that the ongoing inflammation is associated with the activation of the Th1 pathway in this disease. This finding shows further similarities between canine AF and human pCD as it has been widely accepted that Crohn’s disease is driven by an exaggerated Th1 immune response [70]. The second CD4+ T cell subset, which was shown to be involved in the development of IBD is Th17 and related IL-23 cytokine [49]. The importance of this pathway has not been clearly demonstrated so far in the perianal form of CD, and has not been studied in canine AF.

One of the important elements in the pathogenesis of Crohn’s disease is abnormal recognition of microbial molecular motifs by pattern recognition receptors (PRRs). The NOD2 gene encodes a cytosolic PRR recognizing MDP—a peptidoglycan motif common to all bacteria, considered the strongest susceptibility gene associated with Crohn’s disease. Mutations in NOD2 result in inappropriate bacterial recognition and lead to a number of pathological consequences [76]. In dogs, the association of polymorphisms in NOD2 gene with anal furunculosis, although investigated, has not been found [77]. However, the results from one in vitro study suggest that NOD2 function can be disturbed in dogs with AF [45]. Monocytes/macrophages isolated from the blood of dogs with and without AF were exposed to different PRR ligands. It was demonstrated that the response to NOD2 ligand (measured in amount of expressed *TNF*) is significantly lower in cells derived from affected dogs than from healthy ones. Although this difference was not confirmed at the protein level in this study, this issue seems to be worth further investigation. The possible confirmation of an impaired response to NOD2 ligands in dogs with AF would define a new significant pathogenetic factor linking both analyzed diseases.

Metalloproteinases (MMPs) are generally indicated as playing role in the development of diseases associated with significant tissue damage as they are enzymes degrading extracellular matrix (ECM). MMPs were shown to be overactive in many inflammatory diseases including IBD [78]; however, the profile of upregulated MMPs and other related enzymes differ between various conditions. For example, a comparative analysis of MMPs expression in Crohn’s disease (luminal form) and complex diverticulum disease revealed that inflamed mucosa in CD patients displayed higher expression of MMP-2, MMP-9, and MMP-13 than in diverticulitis inflamed mucosa [50]. The gene expression of these three MMPs was evaluated in perianal tissue of dogs with AF (*n* = 40) in comparison to control tissues (from dogs without AF, *n* = 15) [36]. It was shown that lesions in AF canine patients were associated with significant upregulation of *MMP9* and *MMP13*, but not *MMP2*. A study assessing the expression pattern of MMPs in fistulas of patients with Crohn’s disease also demonstrated elevated expression of MMP-9 (using immunohistochemical method) with MMP-2 remaining at similar level in both healthy and fistulizing tissues. At the same time, the expression of natural inhibitors of MMPs (TIMPs) in all the analyzed tissues was low. The presence of MMP-13 was not evaluated in this study [79]. It is difficult to judge about similarities between the MMPs profiles in pCD and AF due to the limited amount of available data, but it seems that the contribution of upregulation of MMP-9 can be common to both conditions. In the context of the use of MSCs in the treatment of perianal fistulas, it should be mentioned that MMP-9 may play a significant role in the impact of MSCs on the surrounding niche. On one hand, it was postulated that MMP-9 (next to MMP-2) can contribute to the suppressive effect of MSCs on T cells [80]. On the other hand, it was shown that MSCs can contribute to MMP-9-mediated epithelial-to-mesenchymal transition (EMT), at least in carcinogenesis [81]. The EMT process is known to be involved in the formation of CD-related fistulas in humans [51]. In dogs with AF, this phenomenon has not been investigated so far.

### 7.3. Genetic Factors

The first studies aimed at identifying genetic determinants associated with canine AF were conducted in 2000s and based on candidate gene approach, in which preselected genes (or haplotypes) were analyzed [43,45,82]. At that time, a relationship between AF and Crohn’s disease was already postulated due to the similarities in the clinical picture and the spectacular response of dogs with AF to the treatment with cyclosporine [30]. Therefore, genes encoding immune-related proteins which were previously identified as genetic determinants in Crohn’s disease were chosen for analysis in these studies. In humans, HLA genes (encoding MHC molecules responsible for the presentation of peptide antigens to T cells) are most polymorphic genes in the whole genome. The association of certain HLA alleles with the incidence of immune-related disorders (including CD) in humans is well established [42]. The frequency and distribution of specific alleles in genes encoding MHC class II in dogs with AF was also assessed. The study was performed on two cohorts of German Shepherds with and without anal furunculosis [43]. The authors found a highly significant association between the DLA-DRB1*00101 allele with the presence of AF indicating the role of abnormal CD4+ T cells activation in response to microbial or self-antigens in the pathogenesis of this disease. This observation suggests a similarity between canine AF and CD since the association of several *HLA-DRB1* alleles with Crohn’s disease has been demonstrated in a number of studies [42]. Such coherence was not shown when analyzing the polymorphisms of the genes encoding the pattern recognition receptors. PRRs are responsible for non-specific recognition of microbial molecular patterns by majority of immune cell types. In humans, *NOD2(CARD15)*, a gene encoding one of cytosolic PRRs, was the first to be linked to Crohn’s disease [83]. Later, this finding was confirmed in many other studies and *NOD2* is currently considered a top gene associated with CD [84]. The absence/presence of certain single nucleotide polymorphisms (SNPs) has also been shown to be associated with the risk of developing perianal fistulas [44]. Another PRR-encoding gene recognized to be related to CD is *TLR4* [85]. In dogs, the association between the occurrence of anal furunculosis with certain variants of several preselected PRRs (including *NOD2* and *TLR4*) has been analyzed in one study including 47 GSD with AF and 100 GSD without AF [45]. No significant correlations have been found.

Currently, the candidate gene approach is no longer popular. The genome wide association studies (GWAS) allow for much better identification of disease-associated genes as they are based on scanning the entire genome or its large fragments without pre-selection. In dogs, the association between genetic variations with anal furunculosis using GWAS was assessed in one study which included 97 affected and 216 healthy German Shepherds from two cohorts [46]. The authors identified 6 non-synonymous (i.e., leading to a changed sequence of amino acids in an encoded protein) SNPs related to the occurrence of anal furunculosis. The regions most significantly associated with the disease were *ADAMTS16* and *CTNND2*. Neither of these genes have been identified as being associated with the occurrence of IBD in humans; however, the *DAP* gene, which is located in the same region of the genome as *CTNND2*, is indicated as associated with ulcerative colitis (though not CD) [46,84]. The authors [46] claim that *CTNND2/DAP* may be a possible shared susceptibility locus between human ulcerative colitis and canine AF; however, it seems that such a statement would require further confirmation.

## 8. Human Mesenchymal Stem/Stromal Cells (hMSCs)—The Rationale behind Their Use in Fistulizing Crohn’s Disease—Potential Mechanisms of Action

Undoubtedly, MSCs are currently considered to be the population with the greatest therapeutic potential in the treatment of perianal fistulas [86]. Cells with MSCs characteristic can be relatively easily isolated from different adult and perinatal tissues and expanded in vitro [87]. Apart from multilineage differentiation capacity, it is well established that MSCs possess immunomodulatory and pro-regenerating properties [88]. Not surprisingly, over the last two decades, researchers and physicians have extensively tested the efficacy of MSC transplantation in the treatment of wide range of immune-mediated diseases, including fistulizing Crohn’s disease [89]. There are several described mechanisms by which human MSCs affect immune cells in vitro. In relation to the already known elements of the Crohn’s disease pathogenesis, the most important seem to be the interactions of MSCs with lymphocytes, macrophages and dendritic cells. First of all, MSCs are well known suppressors of the activation and proliferation of lymphocytes [90,91]. With regard to CD4+ T cell subset, they promote T regulatory cells (Tregs) formation and inhibit differentiation into Th17 cells [92,93]. These properties may be beneficial in restoring the disturbed Treg/ Th17 balance in Crohn’s disease patients. MSCs are also known to affect differentiation of macrophages (Mɸ). They promote Mɸ polarization towards M2-like phenotype [94,95]. MSCs drive macrophages to acquire an enhanced regulatory phenotype with increased secretion of IL-10 and reduced levels of TNF and IL-12 [94]. This activity seems to be an advantage in Crohn’s disease, as the condition is associated with cytokine level disturbances, among which insufficient amount of IL-10 and significant up-regulation of both TNF and IL-12 was reported [96,97]. Another potentially beneficial immunomodulatory effect of human MSCs is affecting the fate of dendritic cells (DCs). DCs have been indicated to play role in the pathogenesis of CD via priming T cells towards Th1/Th17 pathways [97]. MSCs were shown to drive DCs into a phenotype inducing Tregs [98]. The precise mechanisms behind all these immunomodulatory interactions are not fully elucidated. Currently, it is believed they are linked to the expression/secretion of several molecules by MSCs including transforming growth factor beta (TGF-β), indoleamine 2,3-dioxygenase 1 (IDO-1), ligand for programmed cell death protein 1 (PD-L1), cytotoxic T cell antigen 4 (CTLA-4), Fas ligand (FasL), inducible nitric oxide synthase (NOS2), and prostaglandin E2 (PGE2). The immunosuppressive activity of MSCs is induced/enhanced by pro-inflammatory environment, which in vitro is simulated by addition of TNF, IFN-γ or both [99]. Additionally, MSCs secrete several growth factors (some of them in substantial amount), including these with pro-angiogenic activity which can contribute to improved healing. All these data give a very promising image of MSC therapeutic potential in managing Crohn’s disease as well as other inflammatory disorders. However, it should be stated that immunomodulatory properties of MSCs may also contribute to pathological processes. The most relevant seems to be shaping the local tissue environment in the direction favorable for tumor development [100]. Beside suppressing lymphocytes, supporting tumor-associated macrophages and promoting angiogenesis, MSCs also enhance the process of epithelial-to-mesenchymal transition. EMT phenomenon is associated with tumor invasiveness, formation of metastases [81,101], and has been also shown to be the underlying mechanism for the formation of perianal fistulas in Crohn’s patients [102]. Current knowledge in this area indicates that factors such as TNF, TGF-β, IL-13, MMPs, β6-integrin are involved in the EMT process [51]. MSCs are known to mediate their activity via TGF-β. They are also a source of MMPs and, as mentioned before, they were shown to support EMT process in carcinogenesis [81]. It seems that a potential impact of MSCs on EMT progression should also be also considered in research on the perianal cell transplantation in fistulizing CD. Lastly, although many MSC immunomodulating mechanisms have been demonstrated in vitro, their clinical relevance after perianal MSC transplantation in pCD patients has not yet been demonstrated. Conducting parallel studies on animals with a corresponding naturally occurring disease could accelerate the progress in this field.

## 9. Immunomodulatory Properties of Canine Mesenchymal Stem/Stromal Cells

It is known that MSCs can be effectively isolated from canine tissues and multiplicated in vitro [103]. The feasibility of manufacturing and banking of canine MSCs (cMSCs) for veterinary clinical use has also been demonstrated [104]. It was shown that canine MSCs display similar phenotype and differentiation capacity to human MSCs [105]. In two studies, the immunomodulatory properties of canine MSC-derived extracellular vesicles (cMSC-EVs) have been assessed [106,107]. The addition of cMSC-EVs to the stimulated canine peripheral blood mononuclear cells (PBMCs) resulted in a significant decrease in gene expression of pro-inflammatory cytokines (*TNF*, *IL1B*, *IFNG*) with simultaneous increase in the expression of *IL10* and *FOXP3* [107]. The latter effect suggested that cMSC-EVs induced Treg differentiation, and this was more pronounced if MSCs were previously primed with IFN-γ and TNF [107]. The enhanced differentiation of CD4+ T cells into Treg subset in the presence of cMSC-EVs was also demonstrated in cytometric analysis [106]. Moreover, the dose dependent inhibitory effect of cMSC-EVs on the proliferation of the whole PBMCs population as well as on isolated CD4+ T cells was also noted [106]. Furthermore, cMSC-EVs were shown to drive polarization of macrophages into M2 phenotype (measured by changes in gene expression of both M1 and M2 markers) [107]. In another study, the immunomodulatory properties of cMSCs generated from canine induced pluripotent stem cells (ciMSCs) have been evaluated, and compared with cMSCs isolated from adipose tissue and bone marrow [108]. All three populations expressed genes for a variety of immunomodulatory factors, i.e., *NOS2*, *LGALS9* (galectin-9), *TGFB1*, *COX2*, *CXCL8* (interleukin 8) and were responsive to pro-inflammatory priming (measured also at the gene expression level). Overall, canine MSC-EVs as well as cMSCs and ciMSCs were shown to share most important features to human MSCs in regard to immunomodulatory properties. However, the direct comparison of MSCs from both species within one study has not been published so far.

## 10. Conclusions

The accurate assessment of similarities in the pathogenesis of pCD and AF is difficult as the available data are scarce. Clearly, there are significant gaps in our knowledge (especially in canine pathology), i.e., very scant data on the frequency of true fistula formation in dogs with AF, lack of clear evidence confirming that TNF is associated with canine AF, lack of data about the role of EMT process in formation of canine fistulae, or no direct data regarding the activation of Th17 pathway in canine AF. Moreover, some of these gaps will be difficult to fill due to objective reasons, such as a restricted number of canine patients with AF, which hinder conducting research that leads to strong conclusions. It seems that prospective comparative studies with parallel analysis of samples from human pCD and canine AF could define more precisely similarities and differences in the pathology and course of these two diseases, even without including large groups of patients. There are also already defined differences—some of them are obvious, coming from gross anatomy such as the presence/absence of scent anal glands, and some are more sophisticated, such as differences in identified genetic susceptibilities related to genes encoding PRRs, especially *NOD2* (a presence of strong association in humans and lack of association in dogs). Nevertheless, the picture that emerges from the pieces we already have is still very promising. First of all, the pathogenesis of both diseases is multifactorial and main determinants such as the presence of genetic predispositions, clear association with an inappropriate immune response and the relation to intestine microbiota are common. Moreover, numerous more detailed elements of pathogenesis are overlapping in both diseases, such as similar dichotomy of microbiota in affected populations, lymphocytic infiltration of lesions, dominating Th1 pathway, association with certain DRB1 alleles, or the probable role of MMP-9.

Another significant translational value of dogs for human studies (or vice versa) is related to the comparable size of the affected structures as AF refers to large breeds, particularly to German Shepherds. Finally, the properties of canine MSCs described so far appear to be very similar to human ones, suggesting that they may act through similar mechanisms to their human counterparts. Therefore, studying the mechanisms of the immunomodulatory action of MSCs in vivo in dogs may have high translational value to humans. To date, the available data strongly suggest that, with the awareness of certain limitations and shortcomings, the intersection of veterinary and human medicine can help improve the efficacy of cell therapy in the treatment of perianal fistulas. Due to the already-mentioned limited number of canine patients with AF, when organizing veterinary clinical studies to test the effects of cell therapies, multicenter trials would be warranted.

## Figures and Tables

**Table 1:** Chosen aspects of comparative analysis of human perianal Crohn’s disease (pCD) and canine anal furunculosis (AF).

		Humans—Perianal Crohn’s Disease	Ref.	Dogs—Anal Furunculosis	Ref.
**Symptoms**	Association of perianal disease with bowel inflammation	Established. Only about 5% of patients with perianal CD do not have concurrent luminal form of CD	[1]	Probable. 39% of dogs with diagnosed AF do not present histological symptoms of colitis or endoscopic signs of proctitis	[30]
	Perianal symptoms	Fissures, skin tags, ulcers, abscesses, true fistulas with two openings, anal strictures	[37]	Perianal blind sinuses, ulcerations, fistulas communicating with anal sac or anorectum (the incidence of true fistulas with two openings not established)	[20]
**Response to pharmacologic therapy**	Antibiotics	Proven to reduce drainage of perianal fistulas, not to induce healing, recommended as adjunctive therapy	[37]	Efficacy not proven, sometimes used as adjunctive therapy	[20]
	Cyclosporine (CsA)	Efficacy not proven—not recommended	[2]	Proven efficacy—recommended	[38]
	Thiopurine derivatives (i.e., azathioprin)	Moderate efficacy—recommended	[39]	Moderate efficacy based on scant data, sometimes used if CsA therapy is not possible (due to economic reasons or availability problems)	[40]
	Anti-TNF	Proven efficacy—recommended	[41]	Not tested, caninized anti-TNF not available	
**Pathogenesis**	Genetic factors	The role of genes encoding MHC molecules (generally for CD)—confirmed	[42]	the role of genes encoding MHC molecules (DLA-DRB1)—confirmed	[43]
		The role of SNPs in *NOD2* (one of PPRs) (specifically in pCD) - confirmed	[44]	The role of SNPs in chosen PPRs (including NOD2) failed to be confirmed	[45]
		The role of TNF pathway genes (specifically for pCD)—confirmed	[33]	The role of TNF pathway not confirmed	[46]
		The role of other genes associated with cell adhesion, extracelullar matrix, scaffolding proteins or autophagy indicated (specifically for pCD)	[33]	6 non-synonymous SNPs identified to be related to AF. The most significantly associated genes were *ADAMTS16* and *CTNND2*	[46]
	Intestinal microbiota	Association of gut microbiota with pCD confirmed. Dichotomy of microbiome into dysbiotic and non-dysbiotic reported	[47]	Association of gut microbiota with AF confirmed. Dichotomy of microbiome into dysbiotic and non-dysbiotic reported	[48]
	Disturbances in T cell mediated immunity	Association of abnormal T cell response with pCD confirmed. Excessive Th1 response; Excessive Th17 response	[49]	Association of abnormal T cell response with AF confirmed. Excessive Th1 response; role of Th17 response not studied	[31]
	Metallo-proteinases (MMPs)	Elevated expression of MMP-2, MMP-9, and MMP-13 in pCD fistulas	[50]	Elevated expression of *MMP9* and *MMP13* but not *MMP2* in perianal tissue of dogs with AF	[36]
	Epithelial-to-mesenchymal transition	The EMT process confirmed to be involved in the formation of CD-related fistulas	[51]	The association of EMT process and AF not studied	

## Data Availability

Not applicable.

## References

[1] Pogacnik J.S., Salgado G. (2019). Perianal Crohn’s Disease. Clin. Colon Rectal Surg..

[2] Gecse K.B., Bemelman W., Kamm M.A., Stoker J., Khanna R., Ng S.C., Panés J., van Assche G., Liu Z., Hart A. (2014). A global consensus on the classification, diagnosis and multidisciplinary treatment of perianal fistulising Crohn’s disease. Gut.

[3] Adegbola S.O., Pisani A., Sahnan K., Tozer P., Ellul P., Warusavitarne J. (2018). Medical and surgical management of perianal Crohn’s disease. Ann. Gastroenterol..

[4] Panes J., Reinisch W., Rupniewska E., Khan S., Forns J., Khalid J.M., Bojic D., Patel H. (2018). Burden and outcomes for complex perianal fistulas in Crohn’s disease: Systematic review. World J. Gastroenterol..

[5] Cao Y., Su Q., Zhang B., Shen F., Li S. (2021). Efficacy of stem cells therapy for Crohn’s fistula: A meta-analysis and systematic review. Stem Cell Res. Ther..

[6] Panés J., García-Olmo D., Van Assche G., Colombel J.F., Reinisch W., Baumgart D.C., Dignass A., Nachury M., Ferrante M., Kazemi-Shirazi L. (2016). Expanded allogeneic adipose-derived mesenchymal stem cells (Cx601) for complex perianal fistulas in Crohn’s disease: A phase 3 randomised, double-blind controlled trial. Lancet.

[7] Ciccocioppo R., Klersy C., Leffler D.A., Rogers R., Bennett D., Corazza G.R. (2019). Systematic review with meta-analysis: Safety and efficacy of local injections of mesenchymal stem cells in perianal fistulas. JGH Open.

[8] Johnson S., Hoch J.S., Halabi W.J., Ko J., Nolta J., Dave M. (2022). Mesenchymal Stem/Stromal Cell Therapy Is More Cost-Effective Than Fecal Diversion for Treatment of Perianal Crohn’s Disease Fistulas. Front. Immunol..

[9] Lu S., Zhu K., Guo Y., Wang E., Huang J. (2021). Evaluation of animal models of Crohn’s disease with anal fistula (Review). Exp. Ther. Med..

[10] Ryska O., Serclova Z., Mestak O., Matouskova E., Vesely P., Mrazova I. (2017). Local application of adipose-derived mesenchymal stem cells supports the healing of fistula: Prospective randomised study on rat model of fistulising Crohn’s disease. Scand. J. Gastroenterol..

[11] Rivera-Nieves J., Bamias G., Vidrich A., Marini M., Pizarro T.T., McDuffie M.J., Moskaluk C.A., Cohn S.M., Cominelli F. (2003). Emergence of perianal fistulizing disease in the SAMP1/YitFc mouse, a spontaneous model of chronic ileitis. Gastroenterology.

[12] Flacs M., Collard M., Doblas S., Zappa M., Cazals-Hatem D., Maggiori L., Panis Y., Treton X., Ogier-Denis E. (2020). Preclinical Model of Perianal Fistulizing Crohn’s Disease. Inflamm. Bowel Dis..

[13] Bruckner R.S., Nissim-Eliraz E., Marsiano N., Nir E., Shemesh H., Leutenegger M., Gottier C., Lang S., Spalinger M.R., Leibl S. (2019). Transplantation of Human Intestine Into the Mouse: A Novel Platform for Study of Inflammatory Enterocutaneous Fistulas. J. Crohn’s Colitis.

[14] Galipeau J., Sensébé L. (2018). Mesenchymal Stromal Cells: Clinical Challenges and Therapeutic Opportunities. Cell Stem Cell.

[15] Kozawa S., Sagawa F., Endo S., De Almeida G.M., Mitsuishi Y., Sato T.N. (2020). Predicting Human Clinical Outcomes Using Mouse Multi-Organ Transcriptome. iScience.

[16] Kol A., Arzi B., Athanasiou K.A., Farmer D.L., Nolta J.A., Rebhun R.B., Chen X., Griffiths L.G., Verstraete F.J.M., Murphy C.J. (2015). Companion animals: Translational scientist’s new best friends. Sci. Transl. Med..

[17] Hoffman A.M., Dow S.W. (2016). Concise Review: Stem Cell Trials Using Companion Animal Disease Models. Stem Cells.

[18] Ferrer L., Kimbrel E.A., Lam A., Falk E.B., Zewe C., Juopperi T., Lanza R., Hoffman A. (2016). Treatment of perianal fistulas with human embryonic stem cell-derived mesenchymal stem cells: A canine model of human fistulizing Crohn’s disease. Regen. Med..

[19] Arzi B., Webb T.L., Koch T.G., Volk S.W., Betts D.H., Watts A., Goodrich L., Kallos M.S., Kol A. (2021). Cell Therapy in Veterinary Medicine as a Proof-of-Concept for Human Therapies: Perspectives From the North American Veterinary Regenerative Medicine Association. Front. Vet. Sci..

[20] Cain C.L. (2019). Canine Perianal Fistulas: Clinical Presentation, Pathogenesis, and Management. Vet. Clin. N. Am. Small Anim. Pract..

[21] Hermanson J.W., De Lahunta A. (2020). Miller and Evans’ Anatomy of the Dog.

[22] Cohen R., Windsor A. (2013). Anus: Surgical Treatment and Pathology.

[23] Seow-Choen F., Ho J.M.S. (1994). Histoanatomy of anal glands. Dis. Colon Rectum.

[24] McColl I. (1967). The comparative anatomy and pathology of anal glands. Arris and Gale lecture delivered at the Royal College of Surgeons of England on 25th February 1965. Ann. R. Coll. Surg. Engl..

[25] Shabadash S.A., Zelikina T.I. (2002). [Once more about hepatoid circumanal glands of dogs. History of their discovery and reasons for revision the structural and functional data]. Izv. Akad. Nauk. Seriia Biol..

[26] Janssenswillen S., Roelants K., Carpentier S., de Rooster H., Metzemaekers M., Vanschoenwinkel B., Proost P., Bossuyt F. (2021). Odorant-binding proteins in canine anal sac glands indicate an evolutionarily conserved role in mammalian chemical communication. BMC Ecol. Evol..

[27] Budsberg S.C., Spurgeon T.L., Liggitt H.D. (1985). Anatomic predisposition to perianal fistulae formation in the German shepherd dog. Am. J. Vet. Res..

[28] GBD 2017 Inflammatory Bowel Disease Collaborators (2020). The global, regional, and national burden of inflammatory bowel disease in 195 countries and territories, 1990–2017: A systematic analysis for the Global Burden of Disease Study 2017. Lancet Gastroenterol. Hepatol..

[29] Killingsworth C.R., Walshaw R., Dunstan R.W., Rosser E.J. (1988). Bacterial population and histologic changes in dogs with perianal fistula. Am. J. Vet. Res..

[30] Jamieson P.M., Simpson J.W., Kirbyand B.M., Else R.W. (2002). Association between anal furunculosis and colitis in the dog: Preliminary observations. J. Small Anim. Pract..

[31] House A., Gregory S.P., Catchpole B. (2003). Expression of cytokine mRNA in canine anal furunculosis lesions. Vet. Rec..

[32] Day M.J., Weaver B.M.Q. (1992). Pathology of surgically resected tissue from 305 cases of anal furunculosis in the dog. J. Small Anim. Pract..

[33] Kaur M., Panikkath D., Yan X., Liu Z., Berel D., Li D., Vasiliauskas E.A., Ippoliti A., Dubinsky M., Shih D.Q. (2016). Perianal Crohnʼs Disease is Associated with Distal Colonic Disease, Stricturing Disease Behavior, IBD-Associated Serologies and Genetic Variation in the JAK-STAT Pathway. Inflamm. Bowel Dis..

[34] Tsai L., McCurdy J.D., Ma C., Jairath V., Singh S. (2021). Epidemiology and Natural History of Perianal Crohn’s Disease: A Systematic Review and Meta-Analysis of Population-Based Cohorts. Inflamm. Bowel Dis..

[35] de Zoeten E., Pasternak B.A., Mattei P., Kramer R.E., Kader H. (2013). Diagnosis and treatment of perianal Crohn disease: NASPGHAN clinical report and consensus statement. J. Pediatr. Gastroenterol. Nutr..

[36] House A., Catchpole B., Gregory S. (2007). Matrix metalloproteinase mRNA expression in canine anal furunculosis lesions. Vet. Immunol. Immunopathol..

[37] Panés J., Rimola J. (2017). Perianal fistulizing Crohn’s disease: Pathogenesis, diagnosis and therapy. Nat. Rev. Gastroenterol. Hepatol..

[38] Mathews K.A., Sukhiani H.R. (1997). Randomized controlled trial of cyclosporine for treatment of perianal fistulas in dogs. J. Am. Vet. Med. Assoc..

[39] Dejaco C., Harrer M., Waldhoer T., Miehsler W., Vogelsang H., Reinisch W. (2003). Antibiotics and azathioprine for the treatment of perianal fistulas in Crohn’s disease. Aliment. Pharmacol. Ther..

[40] Harkin K.R., Phillips D., Wilkerson M. (2007). Evaluation of Azathioprine on Lesion Severity and Lymphocyte Blastogenesis in Dogs With Perianal Fistulas. J. Am. Anim. Hosp. Assoc..

[41] Shehab M., Alrashed F., Heron V., Restellini S., Bessissow T. (2022). Comparative Efficacy of Biologic Therapies for Inducing Response and Remission in Fistulizing Crohn’s Disease: Systematic Review and Network Meta-Analysis of Randomized Controlled Trials. Inflamm. Bowel Dis..

[42] Mahdi B.M. (2015). Role of HLA typing on Crohn’s disease pathogenesis. Ann. Med. Surg..

[43] Kennedy L.J., O’Neill T., House A., Barnes A., Kyöstilä K., Innes J., Fretwell N., Day M.J., Catchpole B., Lohi H. (2008). Risk of anal furunculosis in German Shepherd dogs is associated with the major histocompatibility complex. Tissue Antigens.

[44] Schnitzler F., Friedrich M., Wolf C., Stallhofer J., Angelberger M., Diegelmann J., Olszak T., Tillack C., Beigel F., Göke B. (2015). The NOD2 Single Nucleotide Polymorphism rs72796353 (IVS4+10 A>C) Is a Predictor for Perianal Fistulas in Patients with Crohn’s Disease in the Absence of Other NOD2 Mutations. PLoS ONE.

[45] House A.K., Gregory S.P., Catchpole B. (2008). Pattern-recognition receptor mRNA expression and function in canine monocyte/macrophages and relevance to canine anal furunuclosis. Vet. Immunol. Immunopathol..

[46] Massey J., Short A.D., Catchpole B., House A., Day M.J., Lohi H., Ollier W.E.R., Kennedy L.J. (2014). Genetics of canine anal furunculosis in the German shepherd dog. Immunogenetics.

[47] Lloyd-Price J., Arze C., Ananthakrishnan A.N., Schirmer M., Avila-Pacheco J., Poon T.W., Andrews E., Ajami N.J., Bonham K.S., Brislawn C.J. (2019). Multi-omics of the gut microbial ecosystem in inflammatory bowel diseases. Nature.

[48] Maldonado-Contreras A., Ferrer L., Cawley C., Crain S., Bhattarai S., Toscano J., Ward D.V., Hoffman A. (2020). Dysbiosis in a canine model of human fistulizing Crohn’s disease. Gut Microbes.

[49] Maggi L., Capone M., Giudici F., Santarlasci V., Querci V., Liotta F., Ficari F., Maggi E., Tonelli F., Annunziato F. (2013). CD4+CD161+ T Lymphocytes Infiltrate Crohn’s Disease-Associated Perianal Fistulas and Are Reduced by Anti-TNF-α Local Therapy. Int. Arch. Allergy Immunol..

[50] Altadill A., Eiró N., González L.O., Junquera S., González-Quintana J.M., Sánchez M.R., Andicoechea A., Saro C., Rodrigo L., Vizoso F.J. (2012). Comparative analysis of the expression of metalloproteases and their inhibitors in resected crohnʼs disease and complicated diverticular disease. Inflamm. Bowel Dis..

[51] Siegmund B., Feakins R.M., Barmias G., Ludvig J.C., Teixeira F.V., Rogler G., Scharl M. (2016). Results of the Fifth Scientific Workshop of the ECCO (II): Pathophysiology of Perianal Fistulizing Disease. J. Crohn’s Colitis.

[52] Ellison G.W. (1995). Treatment of perianal fistulas in dogs. J. Am. Vet. Med. Assoc..

[53] Mathews K.A., Ayres S.A., Tano C.A., Riley S.M., Sukhiani H.R., Adams C. (1997). Cyclosporin treatment of perianal fistulas in dogs. Can. Vet. J..

[54] Hanauer S.B., Smith M.B. (1993). Rapid closure of Crohn’s disease fistulas with continuous intravenous cyclosporin A. Am. J. Gastroenterol..

[55] Present D.H., Lichtiger S. (1994). Efficacy of cyclosporine in treatment of fistula of crohn’s disease. Am. J. Dig. Dis..

[56] Hardie R.J., Gregory S.P., Tomlin J., Sturgeon C., Lipscomb V., Ladlow J. (2005). Cyclosporine treatment of anal furunculosis in 26 dogs. J. Small Anim. Pract..

[57] House A.K., Guitian J., Gregory S.P., Hardie R.J. (2006). Evaluation of the Effect of Two Dose Rates of Cyclosporine on the Severity of Perianal Fistulae Lesions and Associated Clinical Signs in Dogs. Vet. Surg..

[58] Doust R., Griffiths L.G., Sullivan M. (2003). Evaluation of once daily treatment with cyclosporine for anal furunculosis in dogs. Vet. Rec..

[59] Wetwittayakhlang P., Al Khoury A., Hahn G.D., Lakatos P.L. (2022). The Optimal Management of Fistulizing Crohn’s Disease: Evidence beyond Randomized Clinical Trials. J. Clin. Med..

[60] Rovira P., Mascarell L., Bachi P.T. (2000). The Impact of Immunosuppressive Drugs on the Analysis of T-Cell Activation. Curr. Med. Chem..

[61] Archer T., Boothe D., Langston V., Fellman C., Lunsford K., Mackin A. (2013). Oral Cyclosporine Treatment in Dogs: A Review of the Literature. J. Vet. Intern. Med..

[62] Khan K.J., Ullman T.A., Ford A., Abreu M.T., Abadir A., Marshall J., Talley N.J., Moayyedi P. (2011). Antibiotic Therapy in Inflammatory Bowel Disease: A Systematic Review and Meta-Analysis. Am. J. Gastroenterol..

[63] West R.L., Van Der Woude C.J., Hansen B., Felt-Bersma R.J.F., Van Tilburg A.J.P., Drapers J.A.G., Kuipers E.J. (2004). Clinical and endosonographic effect of ciprofloxacin on the treatment of perianal fistulae in Crohn’s disease with infliximab: A double-blind placebo-controlled study. Aliment. Pharmacol. Ther..

[64] Maeda Y., Ng S.C., Durdey P., Burt C., Torkington J., Rao P.K.D., Mayberry J., Moshkovska T., Stone C.D., Carapeti E. (2010). Randomized clinical trial of metronidazole ointment versus placebo in perianal Crohn’s disease. Br. J. Surg..

[65] Thia K.T., Mahadevan U., Feagan B.G., Wong C., Cockeram A., Bitton A., Bernstein C.N., Sandborn W.J. (2009). Ciprofloxacin or metronidazole for the treatment of perianal fistulas in patients with Crohnʼs disease: A randomized, double-blind, placebo-controlled pilot study. Inflamm. Bowel Dis..

[66] Lim S.Z., Chua E.W. (2018). Revisiting the Role of Thiopurines in Inflammatory Bowel Disease Through Pharmacogenomics and Use of Novel Methods for Therapeutic Drug Monitoring. Front. Pharmacol..

[67] Tisdall P., Hunt G.B., Beck J.A., Malik R. (1999). Management of perianal fistulae in five dogs using azathioprine and metronidazole prior to surgery. Aust. Vet. J..

[68] Kalliolias G., Ivashkiv L.B. (2016). TNF biology, pathogenic mechanisms and emerging therapeutic strategies. Nat. Rev. Rheumatol..

[69] Kobayashi T., Momoi Y., Iwasaki T. (2007). Cyclosporine A Inhibits the mRNA Expressions of IL-2, IL-4 and IFN-.GAMMA., but not TNF-.ALPHA., in Canine Mononuclear Cells. J. Vet. Med. Sci..

[70] Guan Q. (2019). A Comprehensive Review and Update on the Pathogenesis of Inflammatory Bowel Disease. J. Immunol. Res..

[71] Kopper J.J., Iennarella-Servantez C., Jergens A.E., Sahoo D.K., Guillot E., Bourgois-Mochel A., Martinez M.N., Allenspach K., Mochel J.P. (2021). Harnessing the Biology of Canine Intestinal Organoids to Heighten Understanding of Inflammatory Bowel Disease Pathogenesis and Accelerate Drug Discovery: A One Health Approach. Front. Toxicol..

[72] Coelho L.P., Kultima J.R., Costea P.I., Fournier C., Pan Y., Czarnecki-Maulden G., Hayward M.R., Forslund S.K., Schmidt T.S.B., Descombes P. (2018). Similarity of the dog and human gut microbiomes in gene content and response to diet. Microbiome.

[73] Day M.J. (1993). Immunopathology of analfurunculosis in the dog. J. Small Anim. Pract..

[74] Bataille F., Klebl F., Rümmele P., Schroeder J., Farkas S., Wild P.-J., Fürst A., Hofstädter F., Schölmerich J., Herfarth H. (2004). Morphological characterisation of Crohn’s disease fistulae. Gut.

[75] Ruffolo C., Scarpa M., Faggian D., Pozza A., Navaglia F., Dʼincà R., Hoxha P., Romanato G., Polese L., Sturniolo G.C. (2008). Cytokine network in rectal mucosa in perianal Crohnʼs disease: Relations with inflammatory parameters and need for surgery. Inflamm. Bowel Dis..

[76] Lauro M.L., Burch J.M., Grimes C.L. (2016). The effect of NOD2 on the microbiota in Crohn’s disease. Curr. Opin. Biotechnol..

[77] House A.K., Binns M.M., Gregory S.P., Catchpole B. (2009). Analysis of NOD1, NOD2, TLR1, TLR2, TLR4, TLR5, TLR6 and TLR9 genes in anal furunculosis of German shepherd dogs. Tissue Antigens.

[78] O’Sullivan S., Gilmer J.F., Medina C. (2015). Matrix Metalloproteinases in Inflammatory Bowel Disease: An Update. Mediat. Inflamm..

[79] Kirkegaard T., Hansen A., Bruun E., Brynskov J. (2004). Expression and localisation of matrix metalloproteinases and their natural inhibitors in fistulae of patients with Crohn’s disease. Gut.

[80] Lavini-Ramos C., Silva H.M., Soares-Schanoski A., Monteiro S.M., Ferreira L.R.P., Pacanaro A.P., Gomes S., Batista J., Faé K., Kalil J. (2017). MMP9 integrates multiple immunoregulatory pathways that discriminate high suppressive activity of human mesenchymal stem cells. Sci. Rep..

[81] Gu J.J., Hoj J., Rouse C., Pendergast A.M. (2020). Mesenchymal stem cells promote metastasis through activation of an ABL-MMP9 signaling axis in lung cancer cells. PLoS ONE.

[82] Barnes A., O’Neill T., Kennedy L.J., Short A.D., Catchpole B., House A., Binns M., Fretwell N., Day M.J., Ollier W.E.R. (2009). Association of canine anal furunculosis with TNFA is secondary to linkage disequilibrium with DLA-DRB1*. Tissue Antigens.

[83] Hugot J.-P., Chamaillard M., Zouali H., Lesage S., Cézard J.-P., Belaiche J., Almer S., Tysk C., O’Morain C.A., Gassull M. (2001). Association of NOD2 leucine-rich repeat variants with susceptibility to Crohn’s disease. Nature.

[84] Garza-Hernandez D., Sepulveda-Villegas M., Garcia-Pelaez J., Aguirre-Gamboa R., Lakatos P.L., Estrada K., Martinez-Vazquez M., Trevino V. (2022). A systematic review and functional bioinformatics analysis of genes associated with Crohn’s disease identify more than 120 related genes. BMC Genom..

[85] Franchimont D., Vermeire S., El Housni H., Pierik M., Van Steen K., Gustot T., Quertinmont E., Abramowicz M., Van Gossum A., Devière J. (2004). Deficient host-bacteria interactions in inflammatory bowel disease? The toll-like receptor (TLR)-4 Asp299gly polymorphism is associated with Crohn’s disease and ulcerative colitis. Gut.

[86] Włodarczyk M., Czerwińska K., Włodarczyk J., Fichna J., Dziki A., Dziki L. (2021). Current Overview on the Use of Mesenchymal Stem Cells for Perianal Fistula Treatment in Patients with Crohn’s Disease. Life.

[87] Hass R., Kasper C., Böhm S., Jacobs R. (2011). Different populations and sources of human mesenchymal stem cells (MSC): A comparison of adult and neonatal tissue-derived MSC. Cell Commun. Signal..

[88] Jiang W., Xu J. (2020). Immune modulation by mesenchymal stem cells. Cell Prolif..

[89] Wu X., Jiang J., Gu Z., Zhang J., Chen Y., Liu X. (2020). Mesenchymal stromal cell therapies: Immunomodulatory properties and clinical progress. Stem Cell Res. Ther..

[90] Dabrowski F.A., Burdzinska A., Kulesza A., Chlebus M., Kaleta B., Borysowski J., Zolocinska A., Paczek L., Wielgos M. (2016). Mesenchymal Stem Cells from Human Amniotic Membrane and Umbilical Cord Can Diminish Immunological Response in an in vitro Allograft Model. Gynecol. Obstet. Investig..

[91] Le Blanc K., Tammik L., Sundberg B., Haynesworth S.E., Ringden O. (2003). Mesenchymal Stem Cells Inhibit and Stimulate Mixed Lymphocyte Cultures and Mitogenic Responses Independently of the Major Histocompatibility Complex. Scand. J. Immunol..

[92] Duffy M.M., Pindjakova J., Hanley S.A., McCarthy C., Weidhofer G.A., Sweeney E.M., English K., Shaw G., Murphy J.M., Barry F.P. (2011). Mesenchymal stem cell inhibition of T-helper 17 cell- differentiation is triggered by cell-cell contact and mediated by prostaglandin E2 via the EP4 receptor. Eur. J. Immunol..

[93] Luz-Crawford P., Kurte M., Bravo-Alegría J., Contreras R., Nova-Lamperti E., Tejedor G., Noël D., Jorgensen C., Figueroa F., Djouad F. (2013). Mesenchymal stem cells generate a CD4+CD25+Foxp3+ regulatory T cell population during the differentiation process of Th1 and Th17 cells. Stem Cell Res. Ther..

[94] Kim J., Hematti P. (2009). Mesenchymal stem cell–educated macrophages: A novel type of alternatively activated macrophages. Exp. Hematol..

[95] Dymowska M., Aksamit A., Zielniok K., Kniotek M., Kaleta B., Roszczyk A., Zych M., Dabrowski F., Paczek L., Burdzinska A. (2021). Interaction between Macrophages and Human Mesenchymal Stromal Cells Derived from Bone Marrow and Wharton’s Jelly—A Comparative Study. Pharmaceutics.

[96] Kamada N., Hisamatsu T., Okamoto S., Chinen H., Kobayashi T., Sato T., Sakuraba A., Kitazume M.T., Sugita A., Koganei K. (2008). Unique CD14+ intestinal macrophages contribute to the pathogenesis of Crohn disease via IL-23/IFN-γ axis. J. Clin. Investig..

[97] Sakuraba A., Sato T., Kamada N., Kitazume M., Sugita A., Hibi T. (2009). Th1/Th17 Immune Response Is Induced by Mesenteric Lymph Node Dendritic Cells in Crohn’s Disease. Gastroenterology.

[98] Li Y.-P., Paczesny S., Lauret E., Poirault S., Bordigoni P., Mekhloufi F., Hequet O., Bertrand Y., Ou-Yang J.-P., Stoltz J.-F. (2008). Human Mesenchymal Stem Cells License Adult CD34^+^ Hemopoietic Progenitor Cells to Differentiate into Regulatory Dendritic Cells through Activation of the Notch Pathway. J. Immunol..

[99] Cuerquis J., Romieu-Mourez R., François M., Routy J.-P., Young Y.K., Zhao J., Eliopoulos N. (2014). Human mesenchymal stromal cells transiently increase cytokine production by activated T cells before suppressing T-cell proliferation: Effect of interferon-γ and tumor necrosis factor-α stimulation. Cytotherapy.

[100] Hass R. (2020). Role of MSC in the Tumor Microenvironment. Cancers.

[101] Liang W., Chen X., Zhang S., Fang J., Chen M., Xu Y., Chen X. (2021). Mesenchymal stem cells as a double-edged sword in tumor growth: Focusing on MSC-derived cytokines. Cell. Mol. Biol. Lett..

[102] Scharl M., Frei S., Pesch T., Kellermeier S., Arikkat J., Frei P., Fried M., Weber A., Jehle E., Rühl A. (2013). Interleukin-13 and transforming growth factor β synergise in the pathogenesis of human intestinal fistulae. Gut.

[103] Carrade D.D., Borjesson D.L. (2013). Immunomodulation by mesenchymal stem cells in veterinary species. Comp. Med..

[104] Luo H., Li D., Chen Z., Wang B., Chen S. (2021). Manufacturing and banking canine adipose-derived mesenchymal stem cells for veterinary clinical application. BMC Vet. Res..

[105] Delfi I., Wood C., Johnson L., Snow M., Innes J., Myint P., Johnson W. (2020). An In Vitro Comparison of the Neurotrophic and Angiogenic Activity of Human and Canine Adipose-Derived Mesenchymal Stem Cells (MSCs): Translating MSC-Based Therapies for Spinal Cord Injury. Biomolecules.

[106] Teshima T., Yuchi Y., Suzuki R., Matsumoto H., Koyama H. (2021). Immunomodulatory Effects of Canine Adipose Tissue Mesenchymal Stem Cell-Derived Extracellular Vesicles on Stimulated CD4+ T Cells Isolated from Peripheral Blood Mononuclear Cells. J. Immunol. Res..

[107] An J.-H., Li Q., Bhang D.-H., Song W.-J., Youn H.-Y. (2020). TNF-α and INF-γ primed canine stem cell-derived extracellular vesicles alleviate experimental murine colitis. Sci. Rep..

[108] Shahsavari A., Weeratunga P., Ovchinnikov D.A., Whitworth D.J. (2021). Pluripotency and immunomodulatory signatures of canine induced pluripotent stem cell-derived mesenchymal stromal cells are similar to harvested mesenchymal stromal cells. Sci. Rep..

